# Editorial: Wnt Signaling at the Plasma Membrane: Activation, Regulation and Disease Connection

**DOI:** 10.3389/fcell.2021.780163

**Published:** 2021-10-25

**Authors:** Gunes Ozhan

**Affiliations:** ^1^Izmir Biomedicine and Genome Center (IBG), Dokuz Eylul University Health Campus, Izmir, Turkey; ^2^Izmir International Biomedicine and Genome Institute (IBG-Izmir), Dokuz Eylul University, Izmir, Turkey

**Keywords:** plasma membrane, Wnt, Frizzled, LRP, lipid raft, membrane order, cancer, regeneration

Wnt signaling pathways constitute evolutionarily conserved signaling pathways that control a wide range of cellular processes including proliferation, migration, differentiation and morphogenesis during development, maintenance of adult tissue homeostasis, and organ regeneration. Thus, it is not surprising that dysregulation of the Wnt pathway has been associated with many human diseases including cancer, congenital malformations, degenerative diseases, and metabolic disorders. At this point, detailed understanding of underlying Wnt-mediated cellular responses is essential for the development of effective therapies against Wnt-related diseases. The plasma membrane (PM) plays a key role in the regulation of cell signaling and thus has been frequently investigated for its molecules as drug targets. Owing to its essential roles in activation of Wnt-receptor complex and initiation of Wnt signaling, the PM has been investigated thoroughly for the regulatory role of its composition and lipid order in pathway regulation. A prominent feature of unhealthy cells such as cancer cells is that they significantly differ from healthy cells with respect to their PM composition and lipid organization. Since the complex interactions of Wnt proteins and their receptors are tightly dependent on the composition and organization of the PM, a better understanding of the molecular mechanisms underlying Wnt-receptor complex formation is essential for identification and testing of novel attractive drug targets for Wnt-related human diseases including cancer. In this Research Topic (RT) with 15 papers, we aimed to collect the review and research articles that provide an overview of the recent developments that enlighten the molecular interaction mechanisms of the Wnt-receptor complex components and pathway modulators as well as how misregulation of these interactions associate with signaling activation and human diseases. We have also included recent scientific progress that we believe that understanding these mechanisms will greatly contribute to the development of novel therapies that target Wnt pathways at the PM.

For this RT, we have received several papers that present detailed analyses of the structure and functional roles of various Wnt ligands, receptors and co-receptors in development and disease. In their review, Suthon et al. have provided a comprehensive view of the mechanistic roles of the non-canonical ligand WNT5B on general development, bone physiology, adipogenesis, pancreas development, heart development, central nervous system (CNS development, physiology of the mammary gland and the lung, hematopoiesis, and the lymphatic system. The review also summarized how Wnt5B is involved in bone diseases including osteoarthritis and osteoporosis, Type II diabetes, neuropathological conditions, chronic obstructive pulmonary disease and various cancers that include breast, pancreas, lung, colorectal, ovary and brain cancers as well as oral squamous cell carcinoma, osteosarcoma, hepatocellular carcinoma, and leukemia. Wei et al. have reviewed the broad biological functions of another Wnt ligand WNT6 in embryonic development. In specific, the authors have focused on decidualization- transformation of the endometrial cells into specialized secretory decidual cells during pregnancy-, kidney development, differentiation of human dental papilla cells, adipogenesis, and osteoblastogenesis. Furthermore, the review provides a detailed overview of the association of WNT6 with various pathologies that include cardiac injury, kidney fibrosis, lung tuberculosis, Rett syndrome, glioblastoma, colorectal cancer, and gastric cancer. In their mini-review on Wnt ligand dependencies in pancreatic cancer, Aguilera and Dawson have discussed the activation of canonical and non-canonical Wnt signaling pathways in pancreatic ductal adenocarcinoma (PDAC). They also review the potential use of therapeutic approaches that functionally antagonize Wnt signaling at the level of ligand secretion or receptor binding. Dhasmana et al., have presented the results of their elegant work on characterization of the role of lipid modifications of the canonical Wnt ligand Wnt3 in membrane organization, secretion, interaction, and signaling activity by exploiting the zebrafish model. By substituting the homologous cysteine and serine residues of zebrafish Wnt3 with alanine, they have revealed that Wnt3 is lipidated at both residues. Lipidation at either of the residues is sufficient for its secretion and proper interaction with the membrane, while the lipid modification at serine is indispensable for receptor interaction and signaling.

Pascual-Vargas and Salinas have elaborately reviewed the molecular function of Frizzled receptors in the mammalian CNS with a special focus on its roles in axon guidance, dendritic morphogenesis, synapse formation and postnatal hippocampus development. The authors have also discussed in detail the post-translational modifications, namely N-linked glycosylation, phosphorylation, and ubiquitination/deubiquitination, of Frizzled receptors regarding their modulation of the receptor function in neuronal connectivity through control of its localization and stability at the PM. Another review by Ren et al. has systematically examined the similarities and divergences between the canonical Wnt co-receptors LRP5 and LRP6 with respect to their expression patterns in human tissues and organ development. They highlight that, in contrast to the high homology between LRP5 and LRP6, they are expressed differentially in diverse tissues and organs in embryonic development and adulthood and that LRP6 is functionally more potent and unique *via* coupling both Wnt with Hippo signaling pathways. The authors have also discussed how Wnt signaling is activated after kidney injury and in renal tissues in diabetes. In their brief research report, Haack et al. have analyzed whether and how the quantitative ratio between membrane receptors and membrane microdomains (rafts) influence LRP6 phosphorylation and WNT/β-catenin pathway activation. By using a computational modeling approach and considering microdomains as single compartments in the membrane, they have found that LRP6 phosphorylation and downstream Wnt signaling activity decreases when the number of raft compartments exceeds the number of pathway specific membrane proteins. This reveals that pathway specific targeting and sorting are critical to restrict the receptor/raft ratio and signaling activation. Jeong and Jho have reviewed activation of LRP6 and its influence on β-catenin-dependent and -independent signaling pathways. They have also discussed in detail how LRP6 dysregulation is associated with diseases including cancer, neurodegeneration, metabolic syndrome, inflammation, and skeletal disease. Shi has thoroughly examined the functional roles of Disheveled in Wnt signaling pathways in gastrulation and neurulation processes by focusing on the vertebrates mouse, *Xenopus*, and zebrafish. The author specifically emphasizes the influence of Disheveled on maternal contribution for axis patterning, cell fate specification and morphogenetic movements.

A comprehensive review by Azbazdar et al. summarizes how membrane composition and lipid order tightly controls formation of the Wnt-receptor complex, its interactions with the pathway modulators and activation of Wnt signaling pathways. Moreover, the study gives a detailed overview of the involvement of the ligands, receptors, coreceptors and extracellular or membrane-bound modulators of Wnt pathways in progression of lung, colorectal, liver, and breast cancers that have been associated with abnormal activation of Wnt signaling.

In their original research, Yong et al. have unraveled the stimulatory role of adiponectin, a secreted protein produced by adipocytes, on the expression of adiponectin receptors as well as β-catenin and MAPK signaling pathways in cementoblasts in the presence of compressive forces. They have also demonstrated that β-catenin is blocked by MAPK inhibition and rescued by adiponectin.

In addition to its well-known roles in development, growth and tissue patterning, Wnt signaling in mammals is necessary for regulation of energy homeostasis. In their elegant review, Azar and Lim have summarized the individual roles of the Wnt pathway components, namely Wnt ligands, β-catenin, destruction complex component APC and transcription factor TCF7L2 in development of metabolic tissues. Moreover, they have discussed the contributions of these components to the whole-body metabolism and potential roles in development of metabolic diseases such as diabetes and obesity.

In their study to understand the role of proliferative stress, Hachim et al. have found that canonical Wnt signaling is activated and required for the asthmatic bronchial epithelial cells to respond to this stimulus. These cells have activated senescence mechanisms in a Wnt-dependent manner. Interestingly, fibroblasts from asthmatic patients were not responsive to inhibition of Wnt signaling.

Finally, Karabicici et al. have shown that the multi-kinase inhibitor regorafenib activates Wnt/β-catenin signaling selectively in hepatoblast-like HCC cell lines. Strikingly, Wnt/β-catenin signaling protects these cells from regorafenib-induced apoptosis. In contrast, long-term regorafenib-treated resistant cells have reduced Wnt/β-catenin activity and enhanced TGF-β activity with a concomitant increase in expression of cancer stem cell markers. Importantly, regorafenib treatment and TGFβ-R1 inhibition have collectively decreased colony formation ability and promoted cell death in resistant spheroids. These results demonstrate that HCC tumors with aberrantly activated Wnt/β-catenin pathway are more resistant to regorafenib, suggesting that regorafenib administration in combination with pathway inhibition can enhance the drug-induced cell death. On the other hand, acquired regorafenib resistance in HCC can be overcome with the combined use of TGF-β pathway inhibitors together with regorafenib.

To conclude, this RT provided us with the opportunity to collect recent data and perspectives from researchers whose studies on Wnt signaling pathways span over a wide range of disciplines. By enabling us to review the recent findings and advances in activation and regulation of Wnt signaling in health and disease, we believe that this RT will greatly contribute to our understanding of pathway mechanisms and the development of new therapeutic approaches for Wnt-related human diseases.

## Author Contributions

GO has written the Editorial manuscript.

## Funding

GO Lab has been supported by the Scientific and Technological Research Council of Turkey (TUBITAK, grant numbers 217Z123, 217Z141, 114C074, 215Z365, 219Z040, and 118C459) and EMBO Installation Grant (IG 3024).

## Conflict of Interest

The author declares that the research was conducted in the absence of any commercial or financial relationships that could be construed as a potential conflict of interest.

## Publisher's Note

All claims expressed in this article are solely those of the authors and do not necessarily represent those of their affiliated organizations, or those of the publisher, the editors and the reviewers. Any product that may be evaluated in this article, or claim that may be made by its manufacturer, is not guaranteed or endorsed by the publisher.

